# CMOS Electrochemical Instrumentation for Biosensor Microsystems: A Review

**DOI:** 10.3390/s17010074

**Published:** 2016-12-31

**Authors:** Haitao Li, Xiaowen Liu, Lin Li, Xiaoyi Mu, Roman Genov, Andrew J. Mason

**Affiliations:** 1 Maxim Integrated Products Inc., 160 Rio Robles, San Jose, CA 95134, USA; 2 Xcellcure LLC., 1 City Place Drive Suite 285, St. Louis, MO 63131, USA; liuxiaowen08@gmail.com; 3 Department of Electrical and Computer Engineering, Michigan State University, East Lansing, MI 48824, USA; lilin02@gmail.com (L.L.); mason@msu.edu (A.J.M.); 4 Apple Inc., 1 Infinite Loop, Cupertino, CA 95014, USA; muxiaoyi@gmail.com; 5 Department of Electrical and Computer Engineering, University of Toronto, Toronto, ON M5S 3G4, Canada; roman@eecg.toronto.edu

**Keywords:** electrochemical, biosensor, amperometry, potentiostat, current readout, impedance spectroscopy

## Abstract

Modern biosensors play a critical role in healthcare and have a quickly growing commercial market. Compared to traditional optical-based sensing, electrochemical biosensors are attractive due to superior performance in response time, cost, complexity and potential for miniaturization. To address the shortcomings of traditional benchtop electrochemical instruments, in recent years, many complementary metal oxide semiconductor (CMOS) instrumentation circuits have been reported for electrochemical biosensors. This paper provides a review and analysis of CMOS electrochemical instrumentation circuits. First, important concepts in electrochemical sensing are presented from an instrumentation point of view. Then, electrochemical instrumentation circuits are organized into functional classes, and reported CMOS circuits are reviewed and analyzed to illuminate design options and performance tradeoffs. Finally, recent trends and challenges toward on-CMOS sensor integration that could enable highly miniaturized electrochemical biosensor microsystems are discussed. The information in the paper can guide next generation electrochemical sensor design.

## 1. Introduction

Quantification of biological or biochemical processes is of utmost importance for many healthcare, biotechnology and research applications. The global demand for biosensors is forecasted to reach $20 billion by the year 2020 [1]. Among the technologies for biosensing [2,3], electrochemical techniques have been widely employed to capture biological recognition events and directly translate results into the electronic information domain. Electrochemical biosensors have been developed to detect a range of biological targets and events, including hybridized DNA [4,5,6], neuron tissue [7,8], bacteria [9,10,11] and enzyme reactions [12,13,14]. Electrochemical biosensors have several advantages in performance and utility: they allow label-free detection, eliminating the time, cost and complexity of use associated with sample labeling techniques; many do not rely on affinity binding and, therefore, permit continuous real-time measurement; and they are well suited for miniaturization to, e.g., wearable platforms. Compared to label-free optical biosensors, electrochemical sensors are also less susceptible to interference from non-specific binding. Compared to traditional optical biosensors, electrochemical biosensors have several advantages in performance and utility: many provide label-free detection, eliminating the time, cost and complexity of use associated with sample labeling techniques; and they are well suited for miniaturization to, e.g., wearable platforms.

The tremendous growth and advances in microelectronics over the past few decades have elevated complementary metal oxide semiconductor (CMOS) integrated circuits technology to a robust and inexpensive electronics platform that is well suited for the implementation of electrochemical instrumentation. With the advances of CMOS-compatible micro-electrodes, a trend exists to build miniaturized biosensors on silicon chips. Compared to traditional benchtop sensors and instrumentation, CMOS biosensors provide lower cost, lower power and smaller size [15]. They also allow the creation of high-density CMOS biosensor arrays, which enable new sensor platforms, such as DNA testing and drug screening [16]. The past 10–15 years have seen a wide range of CMOS electrochemical circuits introduced that impart a variety of instrumentation features and performance capabilities. To better understand the body of work that has been generated and promote future work that improves upon this foundation, this paper overviews electrochemical sensor concepts from an instrumentation point of view and analyzes performance tradeoffs of the CMOS instrumentation circuits that have been reported for electrochemical biosensors. In addition, this paper outlines the opportunity to adapt CMOS instrumentation into integrated microsystems, discusses challenges faced in the process and reviews recent efforts to address these issues. Section 2 categorizes and describes the electrochemical techniques available for biosensors. Existing CMOS instrumentation structures for electrochemical biosensors are then reviewed in Section 3. Section 4 presents on-CMOS sensor integration challenges and reviews approaches for on-chip electrode fabrication and chip-scale packaging.

## 2. Electrochemical Biosensor Techniques

**Figure 1:**
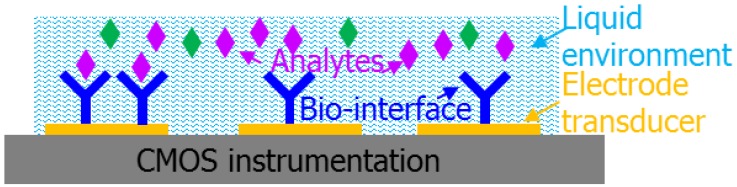
Components of a general integrated electrochemical biosensor.

An electrochemical biosensor can be generally described as consisting of four functional components: analyte, bio-recognition element, transducer and instrumentation, as illustrated in Figure 1. The analyte is the target of interest, such as a protein, DNA, bacteria, etc. The bio-recognition element, also called the bio-interface, imparts the capability for selective recognition of a target analyte. The transducer translates interactions between the analyte and bio-recognition element into an electrical signal. The typical transducer for an electrochemical biosensor is an electrode that translates the flow of ions generated by binding reactions between the analyte and the bio-recognition element into an electrical current or voltage. The instrumentation component is typically comprised of electronic circuitry that captures, amplifies and records biorecognition signals from the transducer. The liquid environment consists of an electrolyte solution that supports the analyte’s biological activity and transports analytes to the transducer.

A wide range of electrochemical techniques can be employed to characterize biosensors or measure their response during biorecognition events. Historically, these techniques were developed to characterize electrode materials and interface reactions, and electrochemists classify them as potentiometry, amperometry, voltammetry, impedance spectroscopy, etc. However, these “ometry” classes do not clearly reflect the sensor’s signaling modality or their instrumentation needs. Therefore, this paper adopts a classification scheme based on the type of response signal generated [17], and this section defines electrochemical sensors as potentiometric, amperometric or impedimetric based on their responses as DC potentials, DC currents or AC signals, respectively. This section also describes field-effect electrochemical biosensors, and it discusses the impact of sensor electrode structure on electrochemical instrumentation.

### 2.1. Potentiometric Biosensor

In traditional potentiometric biosensors, a DC electrical potential is measured when there is no current flow between electrodes. A new approach involves applying a controlled DC current [18] and measuring the resulting potential that is proportional to the analyte concentration. Potentiometric biosensors were popular in the past because of their high selectivity, simplicity and low cost. However, low sensitivity limits their use in many modern applications [19]. The current response in potentiometric biosensors is a combination of the sensor response current and the electrode double layer charging current that is modeled by a double layer capacitance in most electrochemical sensor models. The charging current is frequently large and difficult to remove or filter out and, thus, limits the resolution of potentiometric sensors [18]. Some potentiometric biosensors may involve redox reactions and are called redox-potential biosensors. Redox-potential biosensors have been used for glucose and DNA detection. For more information, the readers are referred to [20].

### 2.2. Amperometric Biosensor

From an instrumentation point of view, biosensors that are stimulated by a DC voltage and generate a DC response current can be categorized as amperometric sensors. The response current is associated with oxidation and/or reduction of an electro-active species involved in the recognition process and relates (ideally directly) to the target analyte concentration. The DC stimulus source can be a constant, step, multi-step or ramp potential applied across the electrode-electrolyte interface and serves as the driving force for the biorecognition process. In the electrochemistry literature, techniques using constant DC stimulus voltages are typically called “amperometry”, while those with DC voltages that vary over time are referred to as “voltammetry”. When the stimulus varies in the pattern of a triangle waveform, it is called “cyclic voltammetry”. Amperometric sensors are commonly used as immunosensors [21], enzyme biosensors [22] and pesticide monitors [23].

### 2.3. Impedimetric Biosensor

Impedimetric biosensors measure the AC electrical impedance of the electrode-electrolyte interface at equilibrium [24]. Impedimetric biosensors take advantage of a change that occurs during biorecognition events in bio-interface characteristics, namely its capacitance and/or resistance. An impedimetric biosensor is created by imposing a small sinusoidal stimulus voltage (or current) of variable frequency and measuring the resulting current (or voltage), whose phase and/or amplitude change contains information regarding biorecognition events. This technique is generally referred to as the electrochemical impedance spectroscopy (EIS) and has been employed in a variety of applications, such as monitoring bilayer lipid membranes [16,25], detecting small molecules of biological relevance [26,27] and measuring cell concentrations [28,29]. Compared to potentiometric and amperometric biosensors, an important advantage of impedimetric biosensors is that the stimulus sinusoidal voltage is small (usually 5–10 mV in amplitude) and does not damage or disturb most bio-recognition layers.

### 2.4. Ion-Sensitive Field Effect Transistor Biosensor

**Figure 2:**
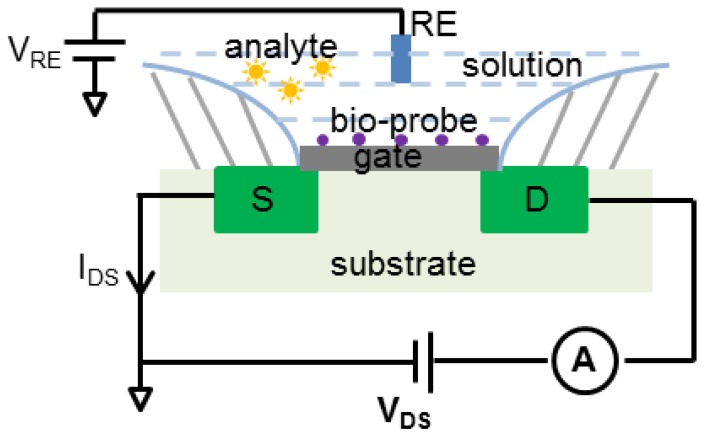
The functional structure of an ion-sensitive field effect transistors (ISFET) biosensor. RE, reference electrode. S is the MOSFET source, D is the drain, and A represents a current meter.

Another method to measure ion concentrations in a solution utilizes ion-sensitive field effect transistors (ISFET). As illustrated in Figure 2, an ISFET operates in a manner similar to a metal-oxide-semiconductor field effect transistors (MOSFET) where the current between source and drain terminals is proportional to the gate voltage that, for an ISFET, is set by reactions during biorecognition events between analytes and bio-recognition immobilized on the gate [30]. ISFET biosensors have been developed for enzyme sensing [31], measuring antigen-antibody bonding reactions [32] and DNA detection [33,34,35]. Immobilization of nanomaterials, such as nanoparticles, nanotubes and nanowires, on the gate has been shown to improve sensitivity [36]. Unlike potentiometric, amperometric and impedimetric biosensors that use an electrode transducer, the ISFET’s transducer is the gate oxide layer. The ISFET response allows the output to be either the gate voltage or source current I_DS_ by fixing one and measuring the other. For example, if I_DS_ is fixed at a value, the gate voltage can be measured using a source follower structure [37]. ISFET output current may be measured by a readout circuit similar to that of an amperometric/impedimetric biosensor. However, it needs more controlled voltages (V_RE_ and V_DS_) and thus requires a different circuit [38]. The remainder of this paper will focus on biosensors with electrode transducers. For more information on the principles, applications and challenges of ISFET biosensors, the reader is referred to [30,39].

### 2.5. Electrode Transducer

**Figure 3:**
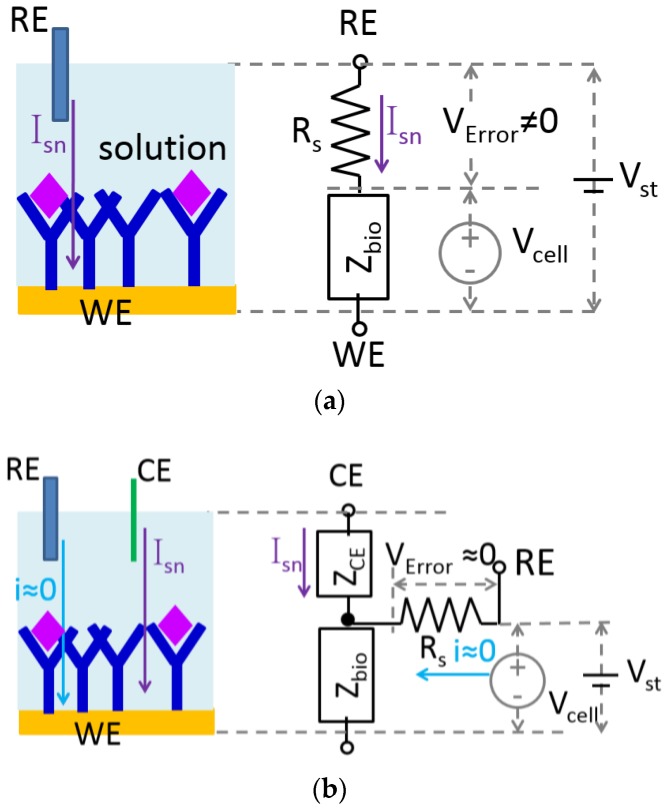
The equivalent circuit model of (**a**) a two-electrode system and (**b**) a three-electrode system. Z_CE_ is impedance between RE and the counter electrode (CE). WE, working electrode.

Electrochemical biosensors employ multiple electrodes to translate biorecognition responses into electrical signals. The electrode where electrochemical reactions of interest take place is called the working electrode (WE). The electrode that provides a stable and known potential is called the reference electrodes (RE). The simplest electrode system utilizes only these two electrodes. In such two-electrode systems, a known stimulus voltage, V_st_, is applied between RE and WE. The resulting potential across the electrochemical cell’s electrode-electrolyte interface, V_cell_, is, ideally, equal to V_st_ throughout the electrochemical reaction. However, as shown in Figure 3a, in addition to the electrode-electrolyte interface, represented by impedance Z_bio_, electrochemical cells inherently possess a solution resistance, R_s_, representing the ion flow path to RE through the electrolyte solution. The sensing current I_sn_ is supplied by RE. As a result, V_cell_ is always less than V_st_ because of the voltage drop, V_Error_, across R_s_, which is commonly referred to as the “IR drop” (I = current, R = resistance). Because the IR drop cannot typically be known in advance and can change with environmental conditions, reaction kinetics, etc., it is generally considered to be an undesirable source of noise/error.

Although R_s_ cannot be entirely removed from an electrochemical cell, the negative effects of IR drop can be eliminated by adding a third electrode, called a counter electrode (CE), to the system. As shown in Figure 3b, in a three-electrode system, I_sn_ is supplied by CE, and in the steady bias state, no current flows through the RE terminal, thus eliminating any IR drop error voltage. As a result, V_cell_ is equal to V_st_, and the reaction potential can be accurately controlled. Because of the performance advantages of eliminating the IR drop effect, three electrode systems are widely used in electrochemical biosensors. Note that potentiometric sensors are generally measured at zero current, and impedimetric sensors generally have small signal AC currents with negligible IR drop. Thus, three-electrode systems are especially useful with amperometric techniques (typically with redox reactions) where larger response currents are generally desired.

Biosensor interfaces with low turnover rates can result in low response currents and low sensitivity. For the redox active bio-recognition element, the redox recycling effect can be utilized to electrochemically magnify the Faradic response current by increasing the mass transport of the redox active species of interest. To utilize redox recycling in biosensors, four-electrode electrochemical systems have been developed [18,40,41]. Four-electrode systems have one RE, one CE and two WEs with the redox recognition element immobilized on both. They are ideally suited for micro-scale interdigitated electrode arrays where the close proximity of WEs enhances redox recycling. Because redox recycling is rarely used and only available with the redox bio-recognition element, four-electrode systems are not very common, and the remainder of this review will focus on popular three-electrode systems.

## 3. CMOS Instrumentation for Amperometric and Impedimetric Biosensors

After electrodes translate biorecognition events into the electrical domain, the signals can then be measured by electronic instrumentation that also provides the biasing signals to drive electrochemical reactions. In modern microelectronics, CMOS circuits have come to dominate the industry due to their low cost and solid performance, especially in low frequency applications, such as biosensing. Consequentially, most of the electrochemical instrumentation developed recently has utilized CMOS technology, and thus, this review will be limited to CMOS electrochemical instrumentation circuits.

Potentiometric biosensor circuits measure the voltage difference at sensor electrodes. A voltage follower circuit topology is generally used to provide a high-impedance interface. Potentiometric circuit examples can be found in [42,43,44]. Because the circuits for potentiometric sensors are relatively simple and not as commonly used in recent biosensors, this paper will focus on circuits for amperometric and impedimetric biosensors that are currently receiving much attention.

**Figure 4:**
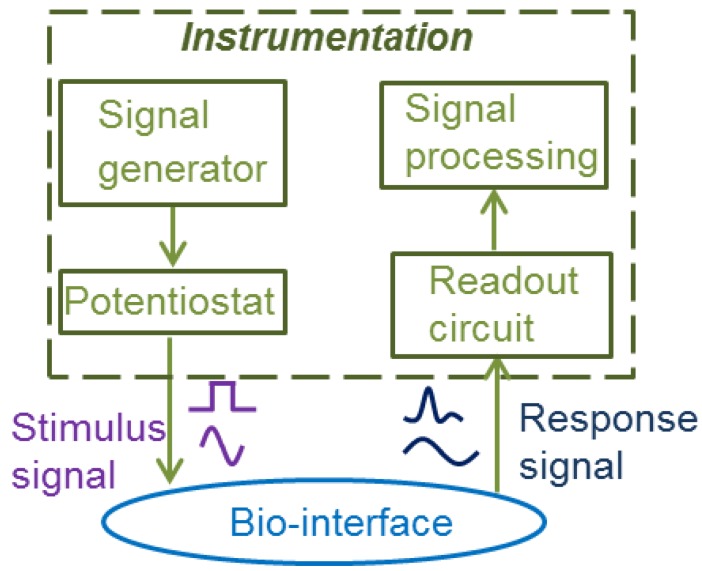
The structure of the instrumentation for electrochemical biosensors.

CMOS electrochemical instrumentation for amperometric and impedimetric biosensors can be organized into four functional blocks, as shown in Figure 4: signal generator, potentiostat, readout circuit and signal processing. The signal generator produces the desired stimulus signal shapes, such as constant, triangle, saw and pulse waveforms. The potentiostat controls the biasing potential of the electrodes. The readout circuit amplifies the sensor response signal and can provide front-end signal conditioning. The signal processing block enhances, e.g., filters, data from the readout circuit. Although a low noise signal generator is necessary for high resolution applications, the signal generator and signal processing blocks do not directly connect to the electrode-electrolyte interface and therefore may be implemented by general circuits. Thus, this review will focus on the potentiostat and readout circuit blocks, which are generally most important to sensor performance.

### 3.1. Potentiostat

In electrochemical sensors, when V_cell_ reaches the analyte’s redox potential, biochemical reactions occurs, so it is critical to have circuits that accurately set V_cell_ during sensor response measurement. The circuit block responsible for establishing V_cell_ on the electrode-electrolyte interface is called a potentiostat.

The first CMOS potentiostat was presented in 1987 [45] and represents the introduction of microelectronics into electrochemical instrumentation, ushering in a new era of miniaturization for high throughput screening, point-of-care diagnostics and implantable device applications. This first circuit was designed for a two-electrode amperometric chemical sensor where a single operational amplifier (OPA) achieves the potentiostat function. Due to the advantages of three-electrode systems compared to two-electrode systems, most of the newer CMOS potentiostats [15,46,47,48,49,50,51,52,53] are designed for three electrodes.

**Figure 5:**
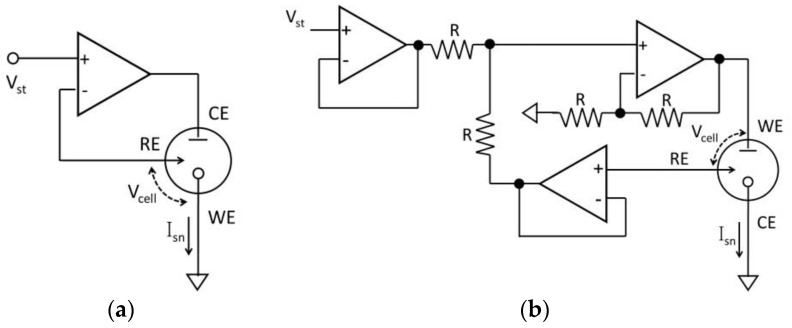
Three-electrode potentiostats with the (**a**) grounded-WE structure and (**b**) grounded-CE structure.

In three-electrode systems, the basic potentiostat configurations are determined by which electrode, WE, RE or CE, is set to the ground reference, often called the analog ground, of the instrumentation circuitry [47]. Configurations that ground RE or CE are electrically equivalent within the potentiostat block leaving the two configurations we will call grounded-WE and grounded-CE. In its simplest form, the grounded-WE potentiostat can be implemented by a single OPA, as shown in Figure 5a. The two inputs of the OPA are connected to stimulus voltage V_st_ and RE, respectively, and WE is connected to analog ground. Because feedback in the OPA forces the OPA inputs to the same potential, the RE voltage (V_RE_) is equal to V_st_. Thus, the electrochemical cell potential, V_cell_, is also equal to the applied V_st_, as desired. The output of the OPA connects to CE, and the electrochemical cell provides a path for the sensing current I_sn_ to the grounded WE, where response current is measured by the readout circuitry.

In the grounded-CE potentiostat, sensor response current is collected from CE, which is connected to the ground. Because the potentials at WE and RE can both vary with time, circuitry is required to ensure that V_cell_ is equal to the applied stimulus, V_st_. The typical three-OPA grounded-CE configuration shown in Figure 5b achieves this goal by setting the voltage on WE, V_WE_, to V_st_ + V_RE_. As a result, V_RE_ − V_WE_ = V_cell_ = V_st_, as desired. Grounded-CE is only rarely used and generally with a specially-designed electrochemical transducer [27]. Because the grounded-CE structure is more complex than the grounded-WE, most reported potentiostats use the grounded-WE structure [15,48,49,50,51,52].

**Figure 6:**
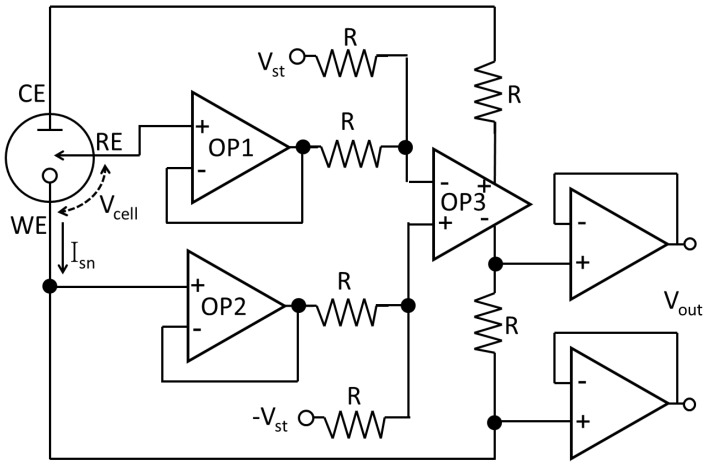
Fully-differential potentiostat (adapted from [46]). OP1–3 are operational amplifiers.

The potentiostat structures in Figure 5 are single-ended potentiostats where the trend to reduce CMOS power supply voltages can drastically limit the range of electrochemical potential applied at V_cell_. To address this problem in low-voltage CMOS, the fully-differential potentiostat shown in Figure 6 was developed to increase the potentiostat output swing [46]. This circuit centers around a fully-differential amplifier, OP3, that dynamically controls the difference between V_WE_ and V_RE_ to be equal to 2V_st_, effectively doubling the V_cell_ output range compared to a single-ended potentiostat. The fully-differential potentiostat topology is very useful for increasing the available V_cell_ potential window when an advanced microelectronics process technology with low supply voltages is desired to reduce power consumption or improve speed.

### 3.2. DC Current Readout Circuitry

In response to a stimulus signal provided by the potentiostat, electrochemical biosensors produce an output signal that is measured by a readout circuit. Because the potentiometric technique is rarely used in biosensors, only readout circuits that measure current responses from amperometric and impedimetric biosensors will be discussed in this paper. From an instrumentation point of view, current readout circuits can be classified as either DC or AC, based on the electrochemical method employed. This section will review DC current readout circuits, while AC current readout circuits are discussed in the next section.

As defined in Section 2, amperometric biosensors are stimulated by a DC voltage, either constant or varying slowly with time, and generate a DC response current. Thus, amperometric biosensors need DC current readout circuits. In general, the amperometric readout circuit should provide a wide current range, high resolution within the bandwidth of interest and allow the measurement of bidirectional currents (sink/source). Numerous CMOS amperometric circuits have been introduced over the past few decades with varying functionality and performance to meet different application demands [15,45,50,51,53,54,55,56,57,58,59,60,61,62,63]. After years of development, the most common structures for CMOS amperometric current readout circuits are resistive, capacitive feedback transimpedance amplifiers (TIA) and the current conveyor. A performance summary of amperometric readout circuits is provided in Table 1.

#### 3.2.1. Resistive Feedback Current Readout Circuits

**Figure 7:**
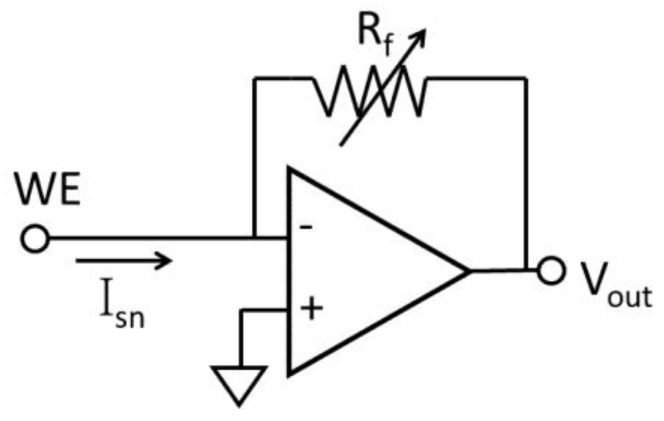
Resistive feedback current readout circuit.

The simplest way to measure a DC response current is to translate the current to voltage through a resistor. As shown in Figure 7, a basic resistive feedback current readout circuit uses an operational amplifier with a feedback resistor R_f_ to convert the biosensor sensing current I_sn_ to a voltage. The sensing current I_sn_ can be calculated by:

(1) $$I_{\mathrm{sn}}=-\frac{V_{\mathrm{out}}}{R_{f}}$$

where V_out_ is the output voltage of the operational amplifier. The dynamic range of the current readout circuitry can be adjusted by changing the R_f_ value. In order to decrease the input referenced current noise, the feedback resistor R_f_ should be large. It has been shown that a readout circuit with resistive feedback can provide a 4-pA noise performance at 10 kHz [61]. However, due to chip area constraints, on-chip R_f_ cannot be very large, and this limits gain. An active feedback structure has been used to decrease the feedback resistor area while achieving pA level noise and a 1-MHz bandwidth using an off-chip filter [62,63]. It has been shown that resistive feedback achieves better noise performance in the high bandwidth than capacitive feedback [64]. This combination of low noise and high bandwidth enables a powerful new platform for biotechnology applications, such as DNA sequencing and ion channel recording.

#### 3.2.2. Capacitive Feedback Current Readout Circuits

**Figure 8:**
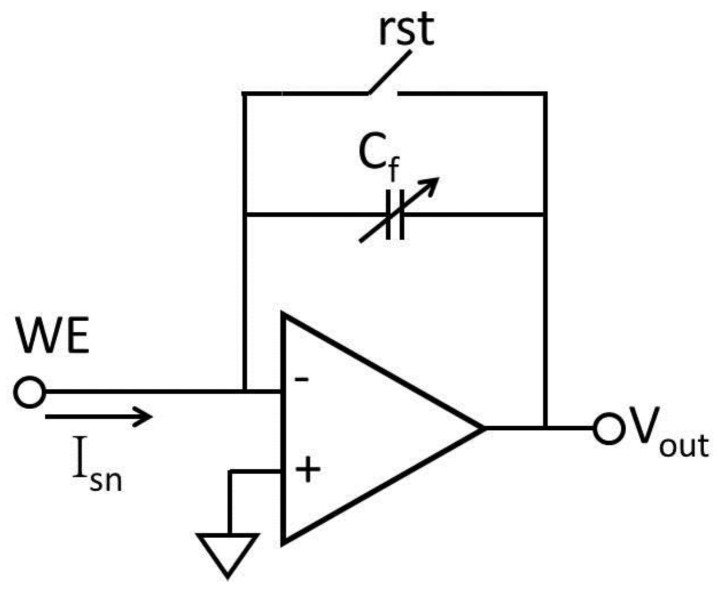
Capacitive feedback current readout circuit.

A drawback of resistive feedback is the thermal noise generated by the feedback resistor, which limits readout resolution. To eliminate this noise source, many current readout circuits with capacitive feedback have been introduced [15,56,57,71,73,74,75,76,77]. It has been shown that, in the low bandwidth, capacitive feedback achieves better noise performance than resistive feedback [64]. The basic capacitive feedback structure shown in Figure 8 is an integrating amplifier that converts the input current to a voltage [74]. The biosensor sensing current I_sn_ can be calculated by:

(2) $$V_{\mathrm{out}}=\frac{1}{C_{f}}\int_{0}^{T}I_{\mathrm{sn}}\mathrm{dt}$$

where C_f_ is the feedback capacitor, T is the integration time and V_out_ is the output voltage of the operational amplifier.

**Figure 9:**
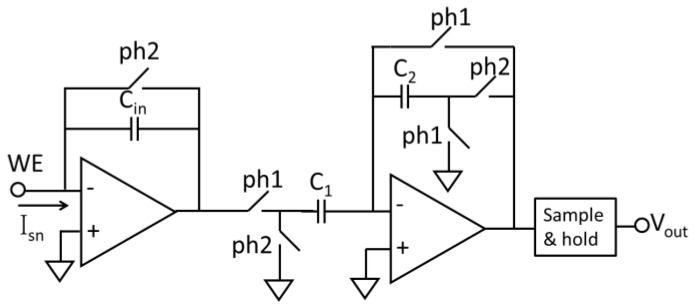
The structure of CDS circuit (adapted from [47]).

The resolution of a capacitive feedback current readout circuit is limited by noise generated in the feedback switch. These noise sources include clock feedthrough, charge injection and KT/C noise and presents as input offset errors. To eliminate this offset error, the correlated double sampling (CDS) technique has been applied to the capacitive feedback readout circuit [15]. The CDS technique can also reduce 1/f noise, which is a significant noise source in DC readout circuitry. The CDS concept involves sampling an input twice and recording the difference of these two samples to reject slow-changing noise/interference. An example CDS circuit is shown in Figure 9, where the two sampling actions are implemented by a set of switches controlled by two inverted phase clocks (ph1 and ph2) [54]. Note that the amplifier should be designed to minimize thermal noise rather than flicker noise because CDS technology can double the thermal noise [61]. Noise aliasing due to sampling can also affect the noise level in CDS circuits [78,79,80]. A current resolution of 10 pA has been achieved using the capacitive feedback structure with CDS technology [54,81].

**Figure 10:**
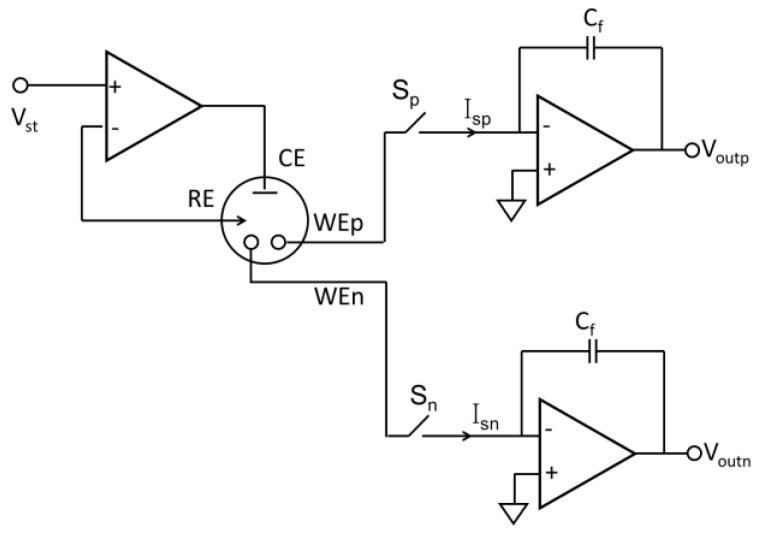
The structure of the pseudo-differential amperometry readout circuit (adapted from [41]).

To reduce the effects of charging currents and currents due to interfering analytes, pseudo-differential capacitive current readout circuits have been developed [48,82]. A schematic of the pseudo-differential readout is presented in Figure 10, where the reading time is controlled by switches S_n_ and S_p_. By using two WEs, the voltage produced by currents from WE_n_ and WE_p_ can be subtracted by a differential amplifier stage (not shown), eliminating nearly all parasitic currents while preserving the analytical signal of interest [48].

#### 3.2.3. Current Conveyor Current Readout Circuits

**Figure 11:**
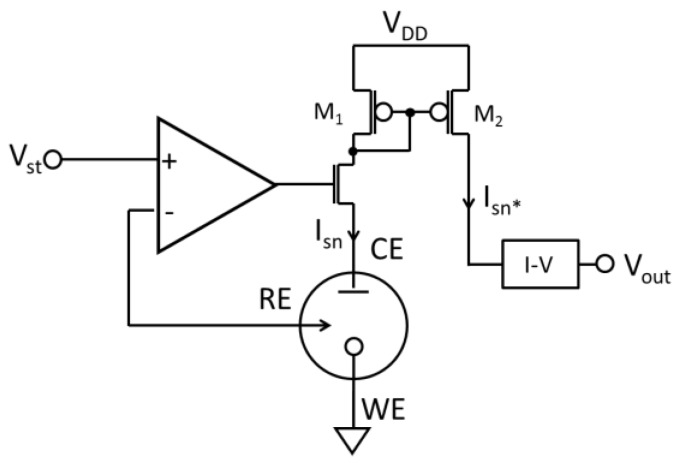
The structure of the current mirror amperometric readout circuit with the potentiostat (adapted from [47]).

The third type of amperometric readout circuit that has been widely reported [47,57,66,69,83,84,85,86] is the current conveyor structure, which uses a front-end current mode amplifier to eliminate noise from the following stages. Figure 11 depicts a typical current conveyor schematic for which the measured biosensor current I_sn_ can be calculated by:

(3) $$\frac{I_{\mathrm{sn}}}{I_{\mathrm{sn}}^{*}}=\frac{{\left(W/L\right)}_{1}}{{\left(W/L\right)}_{2}}$$

where I*_sn_ is the current conveyor output current and W/L is size of current mirror transistors M_1_ and M_2_. The current conveyor circuit performs decoupling and linear operations in current mode in the same way that an operational amplifier performs them in voltage mode. Because no capacitor or resistor is needed for feedback, current conveyors can be very compact [86]. The amplifier in Figure 11 does not need to provide a large output current, allowing the total power consumption of a current conveyor to be very low. Because of these benefits, a current conveyor structure was used in a high throughput biosensor array. Notice that the current conveyor in Figure 11 only measures I_sn_ in one direction. To measure a bidirectional I_sn_, offset current sources or regulated-cascode current mirrors could be employed [66,69].

#### 3.2.4. Digitization Circuits for DC Current Readouts

**Figure 12:**
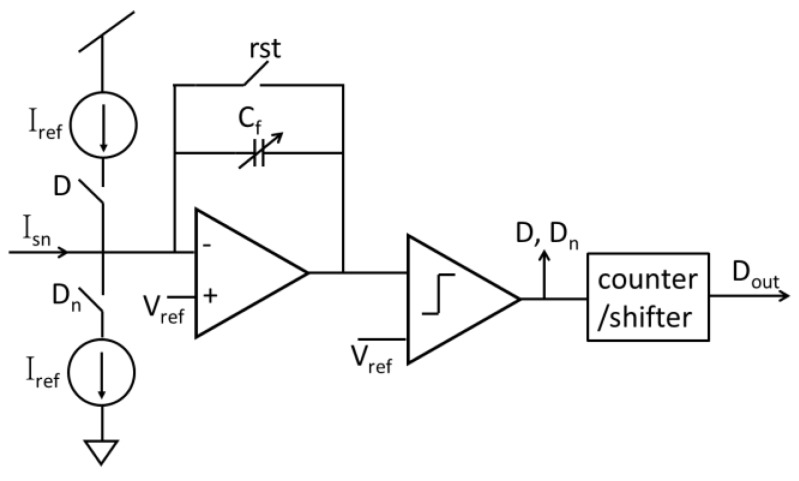
The structure of a first-order incremental current mode Σ∆ ADC circuit for amperometric sensing.

To capture and process sensor signals with digital circuitry, digitization of analog signals is important for features in many biosensor systems permitting efficient analysis and storage of sensor data. Many customized analog-to-digital converter (ADC) structures have been used with amperometric sensors, including sigma-delta (Σ∆) ADCs [56,67,70,71,87], integration ADCs [50,53,57,66,88], current-to-frequency (I-F) ADCs [47,89], successive approximation (SAR) ADC [72] and hybrid topologies [68]. Among these circuits, the incremental first-order Σ∆ ADC structures tend to have the best sensitivity (minimum linear response) of any class of ADC while remaining compact and consuming only microwatts of power. This ADC structure combines the benefits of integration ADCs with the noise shaping of Σ∆ ADCs and achieves superior performance for instrumentation and measurement applications [72]. The structure of a current-mode incremental first-order Σ∆ ADC is shown in Figure 12, where two current sources are used as reference currents in two directions to support a bidirectional input current. Many other efforts to improve Σ∆ ADC performance for amperometric sensors have been reported, including: replacing the traditional comparator with a hysteretic comparator to reduce substrate noise and achieve 50-fA sensitivity [56], using a digital modulation stage to expand the input current range by 32× without significant compromise to power and area [71,87] and reusing sensor capacitance as the amplifier feedback C_f_ to reduce the overall area [70]. Other ADC topologies, such as integration ADC, SAR ADC and I-F ADC, have traditional structures found in textbooks, but have seen limited use in CMOS biosensors because their noise performance is not as good as the incremental Σ∆ ADC.

### 3.3. AC Current Readout Circuitry

**Figure 13:**
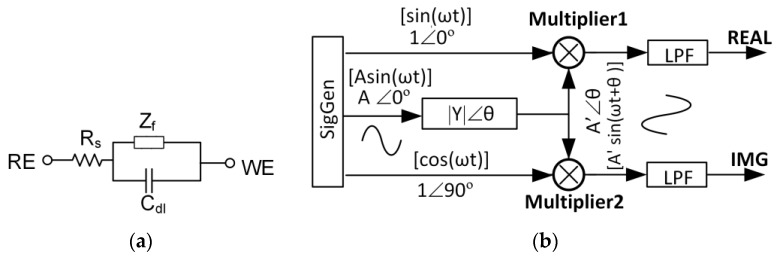
(**a**) Impedimetric sensor circuitry model where C_dl_ represents double-layer capacitance, R_s_ denotes solution resistance and Z_f_ represents Faradic components. (**b**) Frequency response analyzer (FRA) method for extracting transducer impedance/admittance. Both phasor domain and time domain signal expressions are given. ω is the stimulus signal frequency. |Y|∠θ is the phasor form of the transducer admittance. Asin(ω*t*) is the stimulus voltage for the electrochemical transducer. A'sin(ωt + θ) is the output current of the transducer. sin(ωt) and cos(ωt) are the reference signals to extract the real part of imaginary part of the signal. LPF is the low pass filter. REAL and IMG are two orthogonal outputs, representing real and imaginary components of impedance/admittance.

As defined in Section 2, impedimetric sensors are stimulated by a sinusoidal (AC) voltage of known frequency and produce an AC current response that must be measured by readout circuitry. Although materials are often characterized in analytical studies using electrochemical impedance spectroscopy (EIS) over a wide range of sinusoidal stimulus frequencies, in sensing applications, it is more common to use a single frequency or small subset of discrete frequencies where the bio-recognition element has demonstrated a reliable response. Impedimetric sensors are typically modeled by a complex impedance with resistive and capacitive components that can vary with biorecognition events. A typical electrochemical impedimetric sensor interface can be represented by a Randles model circuit, as shown in Figure 13a [18], with model elements defined in the caption. Biorecognition events typically manifest as a phase and/or amplitude shift in the output response relative to the stimulus signal. From this phase-amplitude response, real and imaginary components can be algebraically calculated to extract the sensing parameters of interest from the sensor’s impedance model. If real-time operation or system size is not an application constraint, sensor component extraction can be performed offline. However, several reports have demonstrated that complex impedance extraction can be efficiently implemented within the CMOS readout circuit [16,90,91,92,93,94,95], as highlighted in this section.

#### 3.3.1. Impedance Extraction Algorithms

The real and imaginary components of impedimetric sensors can be extracted in either the digital or analog domain. In the digital domain, the common extraction method is the fast Fourier transform (FFT) algorithm [24]. The FFT algorithm utilizes a broadband stimulus, such as a pulse, and computes the result at all frequency points simultaneously, producing an impedance spectrum. The composite signal source and the computationally-intensive FFT require digital EIS instrumentation to use a digital signal processor (DSP) with extensive computational resources [96]. In the analog domain, real and imaginary components are typically extracted using the frequency response analyzer (FRA) method. FRA processes the response of one frequency point at a time and sweeps over the entire frequency range of interest. Compared to FFT, the FRA method can be realized with simple and compact analog circuits, making it suitable for sensor array microsystems [96]. Therefore, most of the CMOS impedimetric current readout circuits use the FRA method [90,91,92,93,94,97,98].

The operating principle of the FRA method is shown in Figure 13b where a known frequency sinusoid stimulus voltage Asin(ωt) is applied to the electrochemical transducer. The transducer’s response current is defined by A’sin(ωt + θ), where A’ and θ are determined by the transducer’s admittance. By multiplying the response current with two reference signals and then applying low pass filters (LPF), two orthogonal components, real (REAL) and imaginary (IMG), can be extracted, and these signal components are proportional to the real and imaginary components of the transducer’s admittance.

Phasor domain analysis for the transducer response current *A’*∠*θ* in Figure 13, gives:

(4) $$A\prime ∠\theta \left[A\prime \sin\left(\omega t+\theta \right)\right]=A∠0^{0}\cdot \left|Y\right|∠\theta$$

where A’sin(ωt + θ) is the time domain representation of A’∠θ, A’∠0 is the stimulus signal and *|Y|∠θ* is the phasor format of the transducer admittance. From Equation (4), it can be seen that |Y| = A’/A.

The multiplication operations of the FRA circuit give:

(5) $$\{\begin{array}{c} A\prime \sin\left(\omega t+\theta \right)\cdot \sin\left(\omega t\right)=\overset{\mathrm{DC}}{\overset{︷}{\frac{A\prime }{2}\cos\left(\theta \right)}}-\overset{\mathrm{AC}}{\overset{︷}{\frac{A\prime }{2}\cos\left(2\omega t+\theta \right)}}\\ A\prime \sin\left(\omega t+\theta \right)\cdot \cos\left(\omega t\right)=\overset{\mathrm{DC}}{\overset{︷}{\frac{A\prime }{2}\sin\left(\theta \right)}}+\overset{\mathrm{AC}}{\overset{︷}{\frac{A\prime }{2}\sin\left(2\omega t+\theta \right)}} \end{array}$$

where DC and AC labels represent DC component and AC component, respectively. The low pass filters (LPF) in Figure 13 remove the AC components in Equation (5) and generate:

(6) $$\{\begin{array}{c} \mathrm{REAL}=\frac{A\prime }{2}\cos\left(\theta \right)\\ \mathrm{IMG}=\frac{A\prime }{2}\sin\left(\theta \right) \end{array}$$

The transducer’s admittance |Y|∠θ can be calculated by:

(7) $$\{\begin{array}{c} \left|Y\right|=\frac{A\prime }{A}=\frac{\sqrt{{\mathrm{REAL}}^{2}+{\mathrm{IMG}}^{2}}}{2A}\\ \theta =\tan^{-1}\frac{\mathrm{REAL}}{\mathrm{IMG}} \end{array}$$

To simplify signal generator design, the sinusoidal reference signals shown in Figure 13 can be replaced by rectangular waveforms with the same phase of sin(ωt) and cos(ωt) [16]. The higher odd harmonics caused by the rectangular waveform can be eliminated by the LPF.

#### 3.3.2. Impedance Extraction Algorithm Implementation

**Figure 14:**
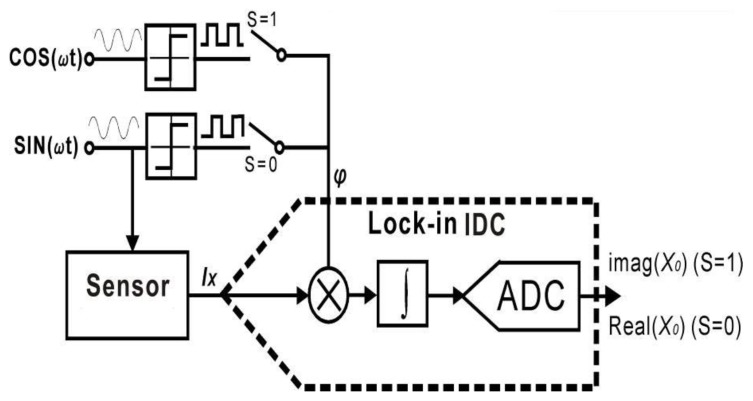
The structure of the lock-in Σ∆ impedance extraction circuit. The circuit extracts the imaginary portion of admittance when S = 1 and the real portion when S = 0 (adapted from [16]).

Several EIS readout circuits have been developed based on the lock-in technique [16,90,91]. A novel structure combining the sigma-delta technique and a lock-in multiplier to support impedance extraction and digitization has been reported [16]. This circuit, shown in Figure 14, shares resources for impedance extraction and digitization to maximize the hardware efficiency, which permits over 100 readout channels within a 3 mm × 3 mm die. The real and imaginary portions are extracted by digital multipliers, which are inherently integrated into the ADC’s integrator, and the high frequency noise is removed by the integrator.

Another impedance extraction circuit applies a transimpedance amplifier to convert the input current into voltage and then extracts impedance using the lock-in technique [92]. This chip is capable of concurrently measuring admittance values as small as 10^−8^ Ω^−1^ with a detection dynamic range of more than 90 dB in the frequency range of 10 Hz–50 MHz [92]. An array of on-chip gold electrodes has been implemented on this chip and used to measure the impedance of the associated electrode-electrolyte interface [92,93].

**Figure 15:**
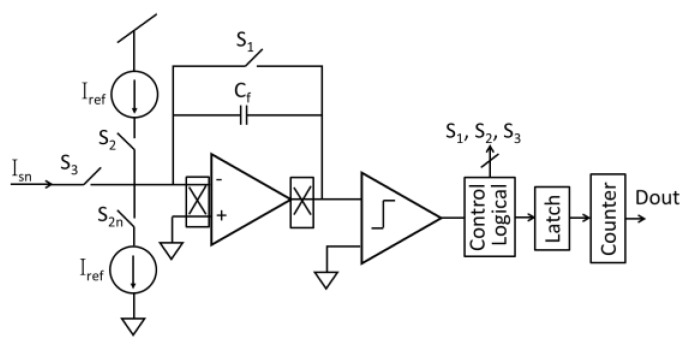
Dual-slope multiplying ADC architecture (adapted from [94]).

To address the bandwidth limitation of the circuit in [16], the impedance spectroscopy DNA analyzer with dual-slope multiplying ADC shown in Figure 15 has been reported [94]. This EIS circuit can extract the analyte impedance in one interrogation cycle. It also includes an on-chip signal generator to produce stimulus waveforms for EIS measurement, albeit with larger area and power consumption.

A common limitation of the impedance extraction circuits discussed above is that they cannot support high frequency stimulus; the analog-multiplier-based circuit [92,93] only supports frequencies up to 50 MHz, and the lock-in ADC [16] and multiplying dual-slope ADC [94] only support frequencies below 10 kHz. To address this issue, a fully-integrated impedance extractor has been reported that can support stimulus frequencies from 20 kHz to 150 MHz by using a down/up conversion architecture [95]. However, this circuit consumes a large amount power, occupies a large area and does not have digital output. The performance of several impedimetric readout circuits is summarized in Table 2.

## 4. CMOS Biosensor Microsystem Integration

**Figure 16:**
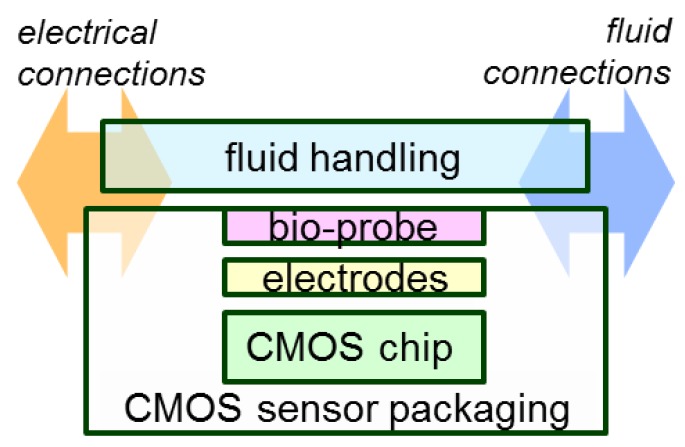
Components in integrating a CMOS biosensor microsystem.

The integration of biosensor interfaces and CMOS detection circuitry has many benefits, including miniaturization, automated detection, high performance and reduced reagent costs. To capitalize on these benefits, a recent trend in CMOS biosensors has been to integrate electrochemical biosensors monolithically with readout circuits to form on-chip measurement microsystems. The interior CMOS high density electrical metal routings can be used to connect electrode arrays to instrumentation circuits, greatly reducing the impact of environmental noise sources and allowing electrical signal connections of high density arrays without the wiring complexity of hybrid off-chip sensors. However, the materials and fabrication processes of CMOS electronics have been developed and selected independently from those of biosensors, leading to a contrast of requirements and compatibility that challenge CMOS biosensor microsystem integration. For example, operation in aqueous biosensing environments is not a requirement for the development of the CMOS electronics. Based on integration efforts to date [99,100,101,102,103,104,105,106,107,108], the components involved in CMOS biosensor microsystem integration are illustrated in Figure 16, where the CMOS chip acts as both the electrochemical instrumentation and the sensor’s physical substrate. Post-CMOS processed sensor electrodes and bioprobes are directly attached to the surface of the CMOS chip and connected to the underlying electronics through thin film planar metal routings at pre-design electrical contact openings. As liquid environments are inherent to biosensors, this integrated sensor requires the interface with fluid handling ranging from simply creating a reservoir over sensors [15] to integrating microfluidic channels to deliver fluids [99,102,103,109]. In addition, packaging is also required that can protect active electronics from the corrosive biosensing liquids. Specifically, the packaging should protect the integrated sensor from potential hazards of chemical corrosion of reactive aluminum CMOS contact pads and electrical shorts through conductive electrolyte fluids. Finally, the entire assembly requires both electrical and fluidic connection to the outside world. Having covered the CMOS electronics above, this section will review efforts to form on-chip biosensor electrodes and implement chip-scale packaging for liquid environments, which are two of the major challenges in CMOS biosensor microsystem integration.

### 4.1. On-Chip Electrodes

In a three-electrode electrochemical system, WE, CE and RE can be implemented with different materials to suit their role in the measurement process. The WE is where bio-probes are immobilized and reactions takes place. Gold or carbon paste is usually used to attach bio-probes through covalent bonding of functional chemical groups of bio-linkers. Platinum is often chosen due to its catalytic role in hydrogen peroxide detection. In recent work, nanomaterials have been conjugated with bio-probes either covalently or non-covalently to amplify the bio-signal. The CE provides a path for reaction current and is expected to be inert during the reaction. The CE is generally much larger than the WE, so that the half reaction at CE can provide sufficient current and does not limit the reaction process at WE. CE is usually made of carbon or a noble metal like gold or platinum. For the RE, whose potential should be stable, independent of the composition of other ions in the analyte and reproducible even after a small electric current flow, a Ag/AgCl, Cl^−^ electrode is preferable. Generally, a Ag rod is coated by barely soluble AgCl and surrounded by a Cl^−^ anion-containing electrolyte reservoir made of liquid-tight coating with a diaphragm or porous membrane. Pseudo-reference electrodes are sometimes used, where only the metal rod is present without the salt or oxide coating and electrolyte reservoir. Use of a pseudo-reference electrode requires specific operation conditions to calculate its potential. In the development of the CMOS-based electrochemical system, to utilize existing mass production techniques driven by semiconductor manufacturing, the electrodes should be implemented in a planar geometry.

**Figure 17:**
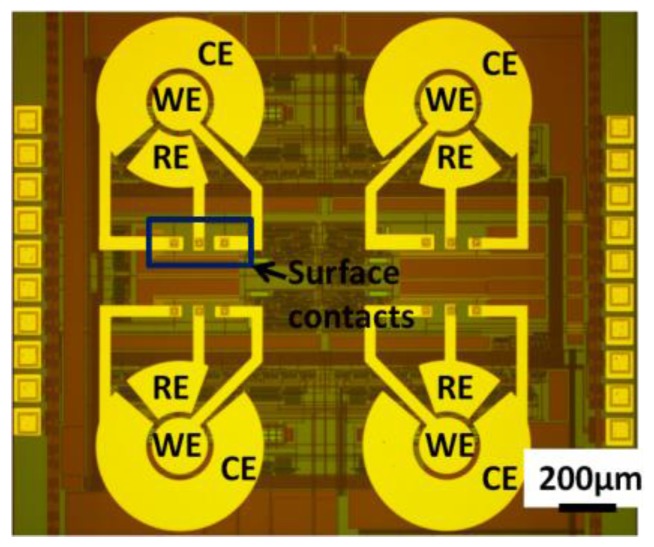
A 2 × 2 thin film planar gold microelectrode array fabricated on a CMOS chip, which includes WE, CE and RE.

Fabricating biosensor electrodes onto the surface of a CMOS chip requires considerations of process compatibility between CMOS functionality and biosensor needs. Furthermore, in low-volume die-level post-CMOS processing, the small size of the CMOS chip (compared to a full wafer) introduces new challenges. For the WE, gold has been electroplated on the aluminum to make an inert electrode, which has affinity for biotargets [110,111]. Alternatively, the top CMOS aluminum layer can be etched away and replaced by another metal electrode material by electroplating, and Pt-black electrodes have been electroplated after aluminum etching for neural recording and stimulation [101]. This post-CMOS process needed no alignment; but the electroplated electrodes have rough surfaces, and the uniform thickness among all of the electrodes was difficult to control. In other work, photolithography-defined planar gold electrodes were deposited on the CMOS surface and connected to circuitry through pre-designed surface contact pads [50,65,112,113]. An example of on-CMOS planar electrodes is shown in Figure 17. These electrodes were flat and uniform, and the geometries of the electrodes could be varied as long as the surface aluminum contacts were properly located on the CMOS. Alternatively, Ti/TiN and tungsten via have been deposited before gold electrode deposition [44]. These gold electrodes are accurately defined, and the surface is smoother than electroplated electrodes.

In contrast to the successes of WE formation on CMOS, the design of a quality planar RE on CMOS for amperometric and potentiometric sensors still remains an open challenge (note that impedimetric sensors typically do not rely on a true reference potential and can accommodate a pseudo-reference electrode). A planar sieve-printed Ag/AgCl quasi-reference electrode on an electrochemical multi-parameter lab-on-chip configuration has been presented on an aluminum oxide ceramic substrate [114]. For a full reference electrode, thin film Ag can be deposited followed by electrochemical chloridization or coverage of sieve-printed AgCl layer. For a long lifetime, a large volume of, e.g., KCl solution should be placed in close proximity in a very limited volume. A proper charge carrier exchange should be established between the solution of the planar reference electrode and the analyte of interest to ensure low resistance of the electrode. Ideally, the loss of KCl and AgCl can be minimized without contamination of the reference element by substances in the solution. To achieve these goals, e.g., hydrogels like agarose have been used to localize KCl and limit the diffusion of the enclosed chloride [115]. The contact between the reference element and the solution could be realized with heterogeneous barriers, which means a hydrophobic layer is facing the outer analyte solution, while a hydrophilic layer is facing inward to reduce contamination [116,117,118]. In other work, a channel-type planar reference electrode has been constructed with a prolonged ion transport path [119,120]. Furthermore, simple integrated glass fibers or filter materials have been used to establish contact to the solution [121]. The surface of the AgCl film has been insulated with a water-repellent layer to slow the loss of chloride [122]. A solid-state reference electrode for an electrochemical system on a microfluidic chip has been reported using electrodeposited nanoporous platinum as the reference electrode [123]. Another solid-state reference electrode was developed utilizing layer-by-layer polymer coating [124].

### 4.2. Chip-Scale Packaging

After the electrodes are fabricated on the CMOS chip, packaging is required before on-chip electrochemical measurements can be performed within a liquid environment. The CMOS chips are most typically wire bonded in a ceramic package, and this scheme has been adapted to perform on-chip measurement by encapsulating the flexible and soft bonding wires to avoid electrical shorts and prevent corrosion caused by electrolyte solutions. These early efforts at integration demonstrate that CMOS biosensor packaging must simultaneously consider both electrical connection of CMOS I/O signals and proper encapsulation of the chip for in-liquid measurements.

In wire bond-based CMOS biosensors, the main goal of encapsulation is to insulate the bonding wires. Recently, many packaging schemes were reviewed and compared with parameters like area efficiency, lifetime, barrier, number of steps, vertical step height, etc. [103]. The packaging material is important because it affects the fabrication process and quality of packaging. Polymer materials have been considered to seal bond wires. PDMS (polydimethylsiloxane), an elastomeric polymer, has been deposited and cured around the CMOS chip to insulate bonding wires [106]. PDMS is widely used as a building material for microfluidic structures and is bio-compatible and inert to most chemicals. Various types of epoxy have also been used to seal bond wires [101,105,125,126,127]. The epoxy was applied around the CMOS chip leaving the chip’s surface exposed. Since epoxy is a liquid material, application of epoxy on bond wires while keeping it off the chip’s surface can be challenging. Epoxies are available in different viscosities, and a proper viscosity can be selected to encapsulate wires with better control. Once cured, epoxy is a hard material compared to PDMS. However, epoxy encapsulation has a reliability issues due to its poor adhesion to the chip substrate and stress imposed on wire bonds. In certain circumstances, the absorption of water can cause cracks in the epoxy and failure of the encapsulation. Another approach was reported using parylene as the encapsulation material [128]. As a thin film polymer material deposited in a low temperature process, parylene can uniformly coat all surfaces and is resistive to chemicals. To expose the electrode area, a micromachining laser source can be used to ablate the parylene during patterning. However, the energy of the laser source is hard to control so that it does not damage the sensing region underneath. Alternatively, an ultrasonic bath can be used to pattern parylene via lift-off, but this could compromise the seal around wire bonds.

Overall, PDMS, epoxy and parylene have shown some degree of success in encapsulation, and selection between them depends on application-specific requirements. PDMS and epoxy are readily available, low cost polymers that can be rapidly formed for encapsulation, but existing techniques to work with these materials lack precision, which can challenge working with smaller chips. On the other hand, parylene can be precisely patterned using a laser source, but requires relatively expensive deposition and laser tools, and it does not provide the mechanical strength of PDMS or epoxy.

**Figure 18:**
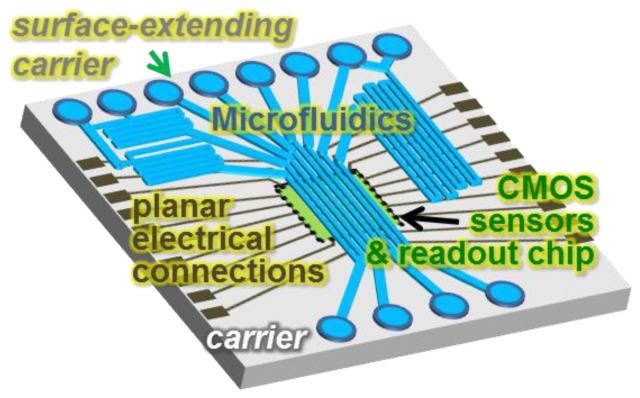
Concept of lab-on-CMOS integration of CMOS biosensors and multichannel microfluidics using a carrier device and planar electrical interconnections.

Recently, to support the integration of CMOS biosensors with microfluidics, an innovative approach called “lab-on-CMOS” was reported using planar metal interconnects and a silicon carrier device [102]. As shown in Figure 18, planar metal interconnects are fabricated on the chip-carrier surface to route CMOS signals to the carrier edge, and finally, a multichannel microfluidic device is attached to the surface. Inspired by chip packaging in wafer level processes [129,130,131,132], the CMOS chip was embedded in a supporting carrier to extend the surface of the chip, so that the increased area could allow the integration of much larger microfluidics. Electrical connections to the chip were realized by planar metal traces deposited by physical vapor deposition. Variations of this approach have recently been reported [103,112]. The lab-on-CMOS platform offers enormous opportunities for integrating CMOS ICs with more complicated multichannel microfluidic structures.

## 5. Conclusions

This paper describes electrochemical biosensors from an instrumentation point of view and reviews the design and performance of CMOS circuits reported for such sensors over the past three decades. This review focuses on two important types of electrochemical biosensors: amperometric and impedimetric, which are classified by their DC and AC responses, respectively. Basic theory and circuit structures are provided; relevant literature is summarized; and performance metrics are reported and discussed. Because the trend in CMOS biosensors is moving toward chip-scale integration, this paper also reviews the fabrication techniques and challenges in the formation of on-chip electrochemical electrodes and on-CMOS biosensor packaging to enable in-liquid measurements. CMOS electrochemical biosensors have a promising future in many applications, and this review paper serves as a guide to future designers who will evolve these sensors even further.

## Figures and Tables

**Table 1:** Performance summary of amperometric current readout circuitry. CDS, correlated double sampling.

	Maximum Input Current	Sensitivity	RMS Noise	Dynamic Range	Power/Channel	Process	Circuit Structure	Target	Target Concentration
JSSC87 [45]	3.5 µA	100 nA	-	31 dB	<2 mW	5 μm	Current conveyor	Glucose	0–24.5 mM
ISSCC94 [53]	40 µA	100 fA	-	172 dB	5 mW	2 μm	Integrator + dual-slope ADC	Cu^2+^	0.5 ppm
JSSC04 [65]	100 nA	10 pA	-	>100 dB	-	0.5 µm	Integration ADC	DNA	-
TCAS06 [56]	100 nA	50 fA	-	60 dB	11 μW	0.5 μm	Semi-synchronous Σ∆ ADC	Bacillus Cereus	0–10^6^ CFU/mL
TCAS06 [66]	50 µA	-	46 pA	120 dB	781 µW	1.2 µm	Integration ADC	Dopamine	0-175 µM
Sensors06 [57]	200 nA	1 pA	-	116 dB	>130 µW	0.5 µm	Integration ADC	KCl	0.01–1 M
TCAS07 [55]	400 pA	20 pA	110 fA	26 dB	1 µW	0.5 μm	Half-amplifier structure; CDS	-	-
TbioCAS07 [67]	0.5 µA	100 fA	-	140 dB	1.3 mW	0.5 µm	Feedback modulation Σ∆ ADC	Dopamine	0–5 µM
JSSC08 [50]	110 nA	~240 pA	-	~53 dB	-	0.25 μm	Dual-slope ADC	DNA	-
TCAS09 [47]	1 µA	1 nA	-	60 dB	70 µW	0.18 µm	Current mirror + I-F ADC ^1^	Glucose	0–38 mM
TBioCAS09 [54]	47 µA	1 pA	-	120 dB	-	0.5 μm	CDS	Alcohol dehydrogenase	0–25 mM
TbioCAS13 [68]	350 nA	24 pA	-	95 dB	188 µW	0.35 µm	I-F + Single slope ADC ^1^	Dopamine	0–80 µM
TCAS13 [69]	350 nA	8.6 pA	0.13 pA	92 dB	4 µW	0.13 µm	Current conveyor	K_3_[Fe(CN)_6_]	0–2 µM
TCAS14 [70]	32 µA	-	-	-	25 µW	2.5 µm	All-CMOS Σ∆ ADC	K_4_[Fe(CN)_6_]	0.1–1.1 mM
TBioCAS16 [71]	16 µA	100 fA	-	164 dB	241 µW	0.5 µm	Input modulated Σ∆ ADC	O_2_	0%–20%
TBioCAS15 [72]	50 nA	-	480 fA	104 dB	12.1 µW	0.18 µm	SAR ADC	Dopamine	0–40 µM
TI-LMP9100x	750 µA	5 µA	-	43.5 dB	33 µW	-	Resistive feedback TIA	Gas/glucose	-

^1^ I-F represents “current-to-frequency”.

**Table 2:** Performance summary of impedimetric readout circuitry.

	Circuit Structure	Frequency Range	Current Range	Process	Power	Target
JSSC09 [16]	Lock-in ΣΔ ADC	10 MHz–10 kHz	78 fA–100 nA	0.5 μm	6 μW	Gramicidin ion channel
TBioCAS10 [92]	analog mixer	10 Hz–50 MHz	330 pA–42 µA	0.35 μm	84.8 mW	DNA/protein
TBioCAS12 [94]	Dual-slope multiplying ADC	0.1 Hz–10 kHz	320 fA–500 nA	0.13 μm	42 µW	Prostate cancer DNA
TCAS05 [98]	Mixer, incremental ADC	-	-	0.8 μm	2.1 mW	Kidney ischemia
ISSCC14 [95]	down/up conversion	20 kHz–150 MHz	-	0.35 μm	0.14 W	-
